# Job Satisfaction and Associated Factors among Anesthetists Working in Amhara National Regional State, Northwest Ethiopia, May 2017: A Multicenter Cross-Sectional Study

**DOI:** 10.1155/2018/6489674

**Published:** 2018-05-02

**Authors:** Demeke Yilkal Fentie, Henos Enyew Ashagrie, Habtamu Getinet Kasahun

**Affiliations:** Department of Anesthesia, School of Medicine, Gondar College of Medicine and Health Sciences, University of Gondar, Gondar, Ethiopia

## Abstract

**Background:**

Job satisfaction is an important determinant of health staff's motivation, retention, and performance. Difficulties in critical decision-making and problems with lack of respect and recognition lead to lower job satisfaction level among anesthetists. It leads to high turnover intention, dropout from the profession, burnout, impaired health status of anesthetists, and lower work performance.

**Objective:**

The aim of this multicenter cross-sectional study was to assess the level of job satisfaction and associated factors among anesthetists working in Amhara National Regional State.

**Methods:**

A multicenter cross-sectional study was conducted from April 1 to June 30, 2017. Ninety-eight anesthetists that were working in Amhara National Regional State Hospitals were involved in this study. The structured questionnaire was scored on five-point Likert scales. Data were analyzed using Statistical Package for Social Sciences version 20. Binary logistic regression was used to measure statistical significance between dependent and each independent variable. Variables with *P* value of ≤0.2 on crude analysis were taken into multivariate analysis, and *P* value 0.05 and 95% CI was used as cut off point.

**Result:**

98 out of 104 participants were involved in this study with a response rate of 94.3%. The overall level of job satisfaction was 46.9%. Anesthetists in academic working position were satisfied with the odds of about 2.3 (AOR = 2.269; CI = 1.137–6.740) compared to those in clinical working position. Anesthetists were least satisfied with coworker relationships (37.8%), work schedule (43.9%), professional opportunity (46.9%), and recognition (49%) while they were most satisfied from their control of responsibility (59.2%), social interaction (55%), and salary and benefits (51%).

**Conclusion and Recommendation:**

Job satisfaction of anesthetists was low, and we suggest that facilitation of professional development, creation of smooth relationship in working environment, increasing the number of anesthetists, and recognition of the anesthesia professional are of paramount importance to increase job satisfaction of anesthetists.

## 1. Introduction

Anesthetists are experts in intensive care, perioperative patient care, acute and chronic pain management, as well as research, and teaching, both at undergraduate and postgraduate levels [1]. Involving in stressful and overloaded working areas results in higher turnover intention, burnout, and job dissatisfaction [2, 3]. Demand-supply imbalance of anesthetists has greatly increased, and they are overworking [4]. Workload and stress due to difficulties in critical decision-making have been shown as problems with the specialty of anesthesia [5]. Anesthetists lack recognition and feedback from patients and even colleagues despite working within a team [5, 6]. All these produce lower job satisfaction that might impair the health of anesthetists their job performance [7].

The WHO in 2013 reported that health-care worker shortages are common globally and the world will be debt of 12.9 million health workers by 2035. Shortage and misdistribution of anesthetists is a social problem in many countries leading to workload, job dissatisfaction, and finally burnout [8]. Lack of recognition from management and lack of public and client awareness towards the role of anesthetists and feedback towards anesthesia have been indicated as causes of low job satisfaction [1, 5, 9].

A study conducted in Switzerland suggests that employees' satisfaction needs to be evaluated, to improve their working condition and to ensure strategies and coping mechanisms to reduce professional stress [5]. Little is known about anesthetists' levels of job satisfaction or workload stress despite their key role in the operating room, ICU, emergency department, and involvement in research and teaching [4]. Lack of job support, long working hours, and challenges in managing life threatening scenarios has been shown to be associated with increased stress and low level of job satisfaction [10].

A study of Netherlands found that job satisfaction as one of the most important and well-investigated work attitudes in organizational behavior, and turnover intention is related to dissatisfaction with job [11]. Another study identified autonomy, collegial relationship, patient-care giver interaction, payment, and availability of resources and educational status as factors that determine job satisfaction of physicians. [12]. One investigation found no difference in satisfaction among men and women anesthetists [13]. A study performed in Kenya showed that inhospitable working condition, limited promotion opportunities, and weak health-care systems result in dissatisfaction and demotivation with work and leaving the country [14]. According to a study in Ethiopia, reasons for dissatisfaction of health-care workers were lack of motivation, inadequate salary, insufficient training opportunities, and inadequate number of human resources, and satisfiers were getting satisfaction from helping others and professional gratification [15]. Control over decision-making and allowing anesthetists to have more influence on their own work pace and flexible work schedule improves working conditions which leads to better job satisfaction and motivation among Switzerland anesthetists [2]. A study conducted in Australia found that high standard of practice and practical aspects of the job were satisfying, whereas poor recognition and long hours were the dissatisfying aspects of the job [6]. Lack of achievement and lack of recognition are identified as dissatisfiers than working conditions and interactions with the seniors [16].

One review commented that improving overall health and improving work satisfaction may decrease burnout among operation theatre team members [17]. Company policies, opportunities for advancement, and the relation between pay and the amount of work are also suggested to be job dissatisfiers among health workers [18]. A meta-analysis conducted in the United Kingdom found that job satisfaction level is an important factor influencing the health of workers [19]. Anesthesia specialty has been identified as a specialty at risk of professional burnout which is associated with poor health including marital difficulties, anxiety, and depression might contribute to alcoholism and drug addiction [7].

Particularly, stress, arising from handling severe illness and death, operating complex equipments, and experiencing a lack of recognition with members of other occupational groups or managers lead to burnout and job dissatisfaction [2, 6, 20]. A study done in Austria showed that one-quarter of anesthetists working in teaching hospitals were at risk for burnout syndrome (characterized by mental and physical exhaustion), and 50% of anesthetists had greater job dissatisfaction (P 0.002) with their job and plan to future dropout from their profession [21].

Job satisfaction of anesthetists is an important key issue for their well being and for improved quality of patient care. The specialty of anesthesia has been classified as high risk for the development of job dissatisfaction. Dropout from the profession due to stress, fatigue, and long working hours is the problem of anesthetists throughout the world. According to the report by Ethiopian Association of Anesthetists, there are a total of 1200 anesthetists serving a population of 100 million in the country showing that there is shortage of anesthesia professionals which may lead to increased workload and job dissatisfaction.

In Ethiopia, the number of anesthetists in intention of turnover, dropout from the profession, and leaving the working area is increasing which is a challenge for the health-care system. One study also showed that job satisfaction is determinant factor for motivation and increased work effectiveness, efficiency, and quality [16]. A cross-sectional study performed in Ethiopia showed that less than half (45.8%) of anesthetists were satisfied with their job [9]. There are limited published data on job satisfaction of anesthetists.

The present study was designed to assess the overall job satisfaction and determine factors affecting the level of job satisfaction among anesthetists which may help health policy makers to implement health policies that focus on incentives, conducive working conditions, fair workloads, and employee retention.

## 2. Methods

Multicenter cross-sectional study was conducted from April to May 2017 in Amhara National Regional State, Northwest Ethiopia.

### 2.1. Study Area

There are five referral and 12 general hospitals in Amhara National Regional State. According to the information from the Amhara Regional Health Bureau, there are about 104 anesthetists working in the region.

### 2.2. Source Population

All anesthetists working in Amhara National Regional State were included in this study.

### 2.3. Study Population

Anesthetists working in Amhara National Regional State who were available during data collection period were included in this study.

### 2.4. Dependent Variable

Level of job satisfaction which was recorded on a five-point Likert scale was the dependent variable.

### 2.5. Independent Variables

Sociodemographic variables including, age, gender, religion, educational status, marital status, years of practice, work-related characteristics, working position, salary, and working hours per week were independent variables.

Job satisfaction subscales such as control responsibility, schedule, coworker interaction, praise and recognition, and social interaction were also incorporated as independent variables.

### 2.6. Operational Definitions

*Job satisfaction*: one's positive perceived emotion on the appraisal of his/her job which is measured by taking the mean (average) score of different 24 items by using five-point Likert scale (from 1 = very dissatisfied to 5 = very satisfied). The overall job satisfaction score was calculated by taking the average (mean) score of all the subscales.

To measure level of job satisfaction of each individual, the mean (average) value of all subscales was calculated. Mean value of subscales was taken as a cut point value to determine whether an anesthetist was satisfied with his/her job or not. As a result, anesthetists for whom score was below mean were considered as dissatisfied and those with mean and above were regarded as satisfied.

*Likert scale*: the sum of response to several Likert items.

*Likert items*: the statement that the respondents are asked to evaluate in the survey.

### 2.7. Data Collection Procedures

Having approval from Institutional Ethical Review Board of University of Gondar, a self-administered questionnaire consisting of sociodemographic variables and 24-item of job satisfaction tool was sent through hand-delivered questionnaire for all anesthetists working in Amhara regional state, and the data were collected by five data collectors within a month after administration of the questionnaires, and it was supervised by the principal investigator.

### 2.8. Data Quality Management

Study participants were oriented on how to fill the questionnaire, and data collection was supervised by the supervisor. The data were cleaned and checked for completeness using Epi info.

### 2.9. Data Processing and Analysis Procedures

Data was coded, entered, and analyzed using SPSS version 20 statistical software. Each item of the job satisfaction was measured by a 5 point Likert scale having a total of 24 items as validated by Tourangeau et al. The reliability coefficient (Cronbach's alpha) of this instrument for our total respondents was 0.88. The 24 items were further divided into seven subscales. Predictor items were also summated accordingly to determine agreement status of the respondent by using computed mean for each sub scale and the higher means score indicating higher satisfaction from the subscales. Descriptive statistics was presented as frequency and percentage.

Using binary logistics regressions model, bivariate and multivariate analyses were employed to determine the association between dependent and independent variables. Variables with *P* value of <0.2 from bivariate analysis were taken to multivariate analysis. *P* values <0.05 were considered statistically significant.

### 2.10. Ethical Consideration

Ethical clearance was obtained from the School of Medicine Ethics Committee of University of Gondar. Written informed consent was obtained from each study participant after clear explanation on what the study entails. Anyone not willing to participate in the study was informed that they have full right not to participate or stop at any time. Confidentiality was ensured by keeping the secrecy of personal identification, completed questionnaires, and results in well secured area.

## 3. Results

### 3.1. Sociodemographic Characteristics

Out of 104 eligible anesthetists working in Amhara National Regional State, 98 responded the self-administered questionnaire, giving a response rate of 94%. Of study participants, 77 (78.6%) were male while 21 (21.4) were females and most of the respondents were unmarried 57 (58.2%). Of study subjects, 61 (62.2%) were in the age group of 25–30 years followed by 9 (9.2%) above 30 years. Of the total respondents, 62 (63.3%) were BSc, 17 (17.3%) MSc, and 19 (19.4%) were diploma anesthetists (Table 1).

### 3.2. Work Related Characteristics

Regarding work experience of anesthetists more than half of them were served about 1–5 years and 6 (6.1%) of them served above 9 years. Of the study participants, 56 (57.1%) were clinical staffs, and 42 (42.9%) were academic staffs.

### 3.3. Job Satisfaction of Anesthetists

Overall, forty six (46.9%) of respondents were satisfied (Table 2). Regarding each subscales, more than half of study participants 58 (59.5%) were satisfied with their control and responsibility at the work place and 54 (55.1%) of anesthetists were satisfied with interaction with patients. Coworker relationship (37; 37.8%), work scheduling (43; 43.9%), professional opportunities, and recognition (48; 49%) were among factors for which less than half of respondents were satisfied.

### 3.4. Factors Associated with Job Satisfaction

Univariate analysis indicated that age of respondents, gender, working position, and medical resource and supply were significantly associated with job satisfaction. The cut point for statistical significance was *P* < 0.20 in crude analysis. All this significant variables in crude analysis were taken into multivariate analysis, and multivariate analysis showed that anesthetists in academic position were about three times more satisfied (AOR = 2.91; CI = 1.20–7.056.740) (Table 3) compared to those in clinical position. Others (age, gender, medical resource, and supply) were not associated significantly with job satisfaction.

## 4. Discussion

Job satisfactions of health-care workers play a vital role in improving the quality of client care. The finding of the present study indicated that less than half (46.9%) of anesthetists were satisfied with their job which is in line with a study conducted in Jimma (Ethiopia) where 45.8% of anesthetists and 41.4% of health workers were satisfied with their job [9, 15].

The result of the present study is inconsistent with the survey conducted in Canada and Nigeria which indicated that 75% and 58.7% of anesthesiologists, respectively, reported overall satisfaction with their job [1, 3]. This might be due to high surgical regard and public image, working in a standard set up, and fair salary and incentives in case of the Canadian and Nigerian studies.

The result of our study showed that anesthetists in academic position were more satisfied compared to those of clinical staffs (AOR = 2.91; CI = 1.20–7.056.740). This might be due to advancing opportunities and difference in salary and incentives. Additionally, professional development might build up the confidence of anesthetists and increase their satisfaction.

In contrast to earlier findings [22, 23] which showed 59.3% and 51.6% of health workers were satisfied, only 37.8% of anesthetists were satisfied to coworker interaction of their respective hospitals. Lack of trained OR assistance and misunderstanding with surgeons might result in lower job satisfaction in the study area. Although our results differ considerably from previous studies, it could be argued that managing critical events, communication problems, [4] and stress might lead to bad reactions to coworkers.

In our study, satisfaction with working schedule was low (43.9%) in accordance with [9, 22, 24] in which heavy work load and less flexible scheduling impaired job satisfaction. This might also be explained by shortage of anesthesia professionals in the study area. This study showed low (46.9%) satisfaction from professional development. This is consistent with a study conducted in Dutch [25] which showed providing sufficient opportunities for learning and growing would produce high job satisfaction level. Scholars reported that acquiring advanced skills and promotion motivate employees and achieve high job satisfaction [26].

The result of the present study showed respondents were less (51%) satisfied with recognition. It could be argued by the reasons that the anesthesia professional is a new specialty in our country and public awareness about the role of anesthetists might be low. Scholars promote that acknowledging employees for their good work is motivator for job satisfaction.

This study revealed that strong control over work and responsibility produces higher job satisfaction (59.5%) in good agreement with [2]. A study conducted in Nigeria suggests that giving enough job freedom and power might help employee to feel own of the results and greater job satisfaction [26]. Supported by the result of a study in Ethiopia, our study indicated that satisfaction with social interaction like helping others and patient outcome was relatively high [9]. This might be due to the fact that anesthetists work to relief pain and suffering diseases of their clients.

## 5. Limitation of the Study

The possible weakness may come from the small sample size, and there is not a special standardized job satisfaction measurement tool for anesthetists, and we modified the job satisfaction scale developed for nurses.

## 6. Conclusion

Job satisfaction of anesthetists working in Amhara National Regional State is low. The only factor associated with job satisfaction was working position. Anesthetists were most satisfied with their control and responsibility and least satisfied with coworker interaction.

## Figures and Tables

**Table 1:** Sociodemographic characteristics of anesthetists working in Amhara National Regional State April to May, 2017 (*N*=98). Data are expressed in frequency and percentage.

Variables	Frequency	Percent
*Gender*		
Male	77	78.6
Female	21	21.4
*Age*		
<25	17	17.3
25–30	61	61.2
31–35	11	11.9
>35	9	9.2
*Marital status*		
Single	57	58.2
Married	41	41.8
*Educational status*		
BSc	62	63.3
Diploma	19	19.4
MSc	17	17.3
*Religion*		
Orthodox	70	71.4
Muslim	16	16.3
Protestant	12	12.3
*Salary in ETB*		
<5000	17	17.3
5000–7000	38	38.8
7000–9000	27	27.6
>9000	16	16.3

**Table 2:** Satisfaction level among participants by job satisfaction subscales in Amhara National Regional State April to May, 2017 (*N*=98). Data are expressed in percentage.

Job satisfaction subscales	Level of satisfaction	
	Satisfied *n* (%)	Dissatisfied *n* (%)
Control and responsibility	58 (59.2)	40 (40.8)
Scheduling	43 (43.9)	55 (56.1)
Social interaction	54 (55.1)	44 (44.9)
Professional opportunities	46 (46.9)	53 (54.1)
Salary and benefits	50 (51)	48 (49)
Praise and recognition	48 (49)	50 (51)
Coworker	37 (37.8)	61 (62.2)
Overall	46 (46.9)	53 (53.1)

**Table 3:** Multivariate analysis for predictors of job satisfaction of anesthetists, Amhara National Regional State April to May, 2017 (*N*=98). Data are expressed in COR and AOR.

Independent predictors	Job satisfaction		COR (95% CI)	AOR (95% CI)
	Satisfied	Dissatisfied		
*Gender*				
Male	39	38	2.05 (0.74–5.64)^∗^	
Female	7	14	1	^∗∗∗^
*Age*				
<25	9	8	1	^∗∗∗^
25–30	23	38	0.53 (0.18–1.59)	
31–35	6	5	1.06 (0.23–4.88)	
>35	8	1	7.11 (0.72–6.85)^∗^	
*Marital status*				
Single	24	33	1.23 (0.55–2.76)	
Married	16	25	1	^∗∗∗^
*Working position*				
Academic	25	17	2.45 (1.08–5.56)^∗^	2.91 (1.20–7.05)^∗∗^
Clinical	21	35	1	1
*Working hour/week*				
≤50	14	13	2.38 (0.83–6.83)	
51–70	11	8	2.62 (0.81–08.43)	
71–90	7	13	1.56 (0.49–4.90)	
>90	8	24	1	^∗∗∗^
*Educational status*				
MSc	5	12	2.66 (0.67–10.58)	
BSc	25	37	2.4 (0.75–7.62)	
Diploma	10	9	1	^∗∗∗^
*Salary*				
<5000	9	8	1	^∗∗∗^
5000–7000	12	26	0.64 (0.20–2.04)	
7000–9000	11	16	0.71 (0.25–2.40)	
>9000	8	8	1.14 (0.29–4.50)	
*Work experience*				
1–3	23	33	1	^∗∗∗^
4–6	11	21	1.17 (0.49–2.81)	
7–9	1	1	1.33 (0.07–22.41)	
≥10	5	3	4.00 (0.74–21.58)	
*Resource and supply*				
Inadequate	22	33	1	^∗∗∗^
Adequate	24	19	1.89 (0.84–4.25)^∗^	

^∗^ *P*  value < 0.2; ^∗∗^*P* < 0.05 and ^∗∗∗^did not appear.

## Abbreviations

ETB: Ethiopian birr
ICU: Intensive care unit
JS: Job satisfaction
WHO: World Health Organization
UOG: University of Gondar.
